# Global Challenges of Being a Strength Athlete during a Pandemic: Impacts and Sports-Specific Training Considerations and Recommendations

**DOI:** 10.3390/sports8070100

**Published:** 2020-07-14

**Authors:** Christopher Latella, G. Gregory Haff

**Affiliations:** 1 Centre for Exercise and Sports Science Research (CESSR), School of Medical and Health Sciences, Edith Cowan University, Joondalup 6027, Australia; g.haff@ecu.edu.au; 2 Neurophysiology Research Laboratory, Edith Cowan University, Joondalup 6027, Australia; 3 Directorate of Psychology and Sport, University of Salford, Salford, Greater Manchester M5 4WT, UK

**Keywords:** powerlifting, weightlifting, COVID-19, resistance training, detraining, injury, performance

## Abstract

The ongoing global pandemic brought about by Coronavirus II (SARS-Cov-2 or COVID-19) has caused an ongoing cessation of sporting competitions and training facility closures. This is a fundamental challenge for amateur and elite sporting professionals. Although recommendations have been provided for team-sport athletes to maintain general and sport-specific conditioning, these methods are often not optimal for strength athletes (i.e., powerlifting (PL) and weightlifting (WL)) due to the unique and narrow set of performance requirements posed by these sports. The purpose of this review is to provide evidence-based information and recommendations and highlight potential strategies and approaches that may be used by strength (PL and WL) athletes during the current global crisis. Collectively, we provide evidence from resistance training literature regarding the loss of muscle strength, power and mass, minimum training frequencies required to attenuate such losses and training re-adaptation. Additionally, we suggest that time off training and competition caused by ongoing restrictions may be used for other purposes, such as overcoming injury and improving movement quality and/or mobility, goal setting, psychological development and emphasizing strength sports for health. These suggestions are intended to be useful for coaches, strength athletes and organizations where existing training strategies and recommendations are not suitable or no longer feasible.

## 1. Introduction

The recent and ongoing pandemic caused by the outbreak of the severe acute respiratory syndrome known as Coronavirus II (SARS-Cov-2 or COVID-19) in late 2019 has caused a major shift in the global way of life. For many affected countries, this has had severe negative impacts on multiple domains including work, travel, leisure activity and national economies. For sporting organizations, restrictions have forced a temporary but ongoing cessation of major events and competitions worldwide [1,2,3,4]. Moreover, these restrictions have extended to the closure of training facilities, including private and commercial gymnasiums. These closures present a fundamental problem for general population health, as well as amateur and elite sporting professionals [5]. Although various strategies are in place in an attempt to attenuate the spread of the virus, and an easing of restrictions is in sight for some countries, this is not the case for all, and the potential for future outbreaks or continued social distancing recommendations is likely to present an ongoing problem for the foreseeable future.

Recently, several research articles have sought to provide recommendations for general population health [6] and fitness [7,8] during this pandemic. Additionally, suggestions have also been provided for athletes [9] and, in particular, team-sport athletes to maintain health [10], body composition, routine, physical conditioning and a safe return to training amongst others [11]. From a physical performance perspective, team-sport athletes and those involved in multidisciplinary sports (e.g., those requiring proficiency across multiple physiological and physical performance domains) may be able to maintain general, and some degree of sport-specific, fitness through methods such as high-intensity interval and circuit-based training. However, these conditioning methods are not suitable for strength (i.e., powerlifting (PL) and weightlifting (WL)) athletes due to the unique set of performance requirements posed by these sports [12,13]. Specifically, PL and WL athletes practice a very confined set of sport-specific strength movements under moderate-to-high loads during training which directly transfers to competition [12,13,14]. These include the squat, bench-press and deadlift for PL and snatch and clean and jerk for WL amongst other movement derivatives and accessory exercises. The routine practice of these skills and ongoing neuromuscular development can allow lifts exceeding 3–5 times body weight for elite PL athletes [15] and 2–3 times body weight in elite WL athletes (derived from Croucher [16]). Thus, the need for specific equipment and high amounts of external load to train effectively can present a unique problem in the current global climate.

Despite obvious constraints and ongoing restrictions, there is potential to mitigate substantial losses in sport-specific capacity for strength athletes. Moreover, the time where access to training facilities and equipment is limited or non-existent may be used to focus on other areas crucial to long-term athletic physical and mental development. These may include implementing strategies to overcome persistent injury, improving mobility, goal setting and using psychological training strategies to improve future competitive performance. The benefits of participation to overall health and community should also be considered at the individual and organizational level. Therefore, the purpose of this review is to highlight the impact of the current global situation on strength sports, provide evidence-based information and pose potential strategies and approaches that may be adopted by strength (e.g., PL and WL) athletes. The suggestions and recommendations are intended to be particularly useful for coaching professionals, strength athletes and organizations where existing physical training and conditioning suggestions and scheduled competitions may not be suitable or are not currently feasible. 

## 2. Impact of the Global Pandemic on Strength Sport Participation and Competitions

**Figure 1:**
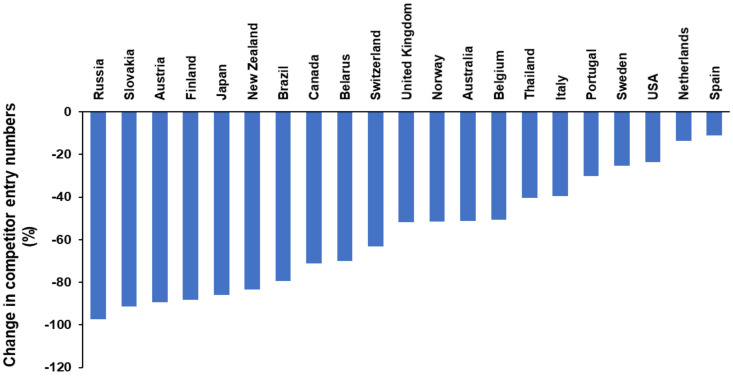
Displays the estimated percentage reduction in competitor entries based on country from the 1 January 2020 to 2 May 2020 compared to same dates in 2019 (data extracted from [21]). Countries where results were not available for both years or were deemed incomplete after manual inspection were omitted.

The impact of the current and ongoing global situation on strength sports at the local, national and international level is now being recognized. Although in some countries sport participation and competition is beginning to resume, this has resulted in significant disruption to date. For example, in Australia, there have only been 40 Australian Weightlifting Federation sanctioned competitions (1 January 2020–31 May 2020) compared to 76 competitions during the same time period in 2019 [17]. A similar reduction has been observed for British Weightlifting with approximately 23 competitions held up to 31 May for 2020, compared to 53 competitions up until the same date in 2019. For national events Japan recorded 5 competitions (1 January 2019–31 May 2019) compared to 1 competition for the same dates in 2020. The reduction is also evident in the sport of PL. Specifically, there have only been 11 listed Powerlifting Australia sectioned competitions this year (1 January 2020–31 May 2020) compared to 37 at the same time last year [18]. Additionally, a reduction in competitions has also been observed by the United States Powerlifting Association with 135 competitions held until 31 May 2019 compared to 89 competitions up until the same date in 2020 [19]. Although similar information is not readily available from all countries, this trend is likely to be similar across organizations. Based on the evidence thus far, it is also likely that the total number of PL competitions during 2020 will be significantly less than the 76 competitions that occurred in 2019 [20], and importantly, this observation is unlikely unique to Australia. In fact, it appears that the impact is widespread across all counties (refer to Table 1). For example, documented global competitor entries based on PL competition data [21] reports a total of 27,303 individual competitor entries across all federations between 1 January 2020 and 2 May 2020. This is in comparison to the 46,378 individual competition entries recorded up until the same time period in 2019, corresponding to an approximated 41% reduction. In a number of countries, the observed reduction in competitor entries has been upwards of 80% when compared to 2019 (Refer to Figure 1). This finding likely stems from multiple factors such a number of organized competition meets being postponed or cancelled. It may also result from a reduced number of athletes choosing to compete, especially during the early part of the year when COVID-19 was apparent, but sporting restrictions were not implemented as yet.

## 3. Training Cessation Effects on Muscle Strength, Power and Mass

For PL and WL athletes, movement-specific strength and power is of utmost importance for competitive success. Thus, attenuating loss of neuromuscular capability during this period is essential to ensure performance is not compromised when competition resumes. When interpreting the available resistance training research, it appears that strength may be partially or completely maintained in the short term (e.g., up to 3 weeks) [22] but will be compromised after 4 weeks (e.g., surfing athletes) [23] and begin to decay in team-sport athletes [24] and/or be substantially lost after 5 weeks without training in physically active males [25]. Tran et al. [23] also noted a reduction in athletes’ sensorimotor ability which may be an important consideration where technical and/or skillful actions are required (e.g., complex WL movements). Further, Izquierdo et al. [26] demonstrated reductions in maximal strength and power of 6% to 9% and 14% to 17% in the upper- and lower-limbs, respectively, following 4 weeks of a complete cessation of training in physically active men. While both muscular strength and power decreased during this 4-week time period, the reduced ability to express high power outputs were more pronounced. Although the exact reasons for these responses are unclear, it seems reasonable to assume that more substantial reductions may occur in explosive complex multi-joint movements requiring a high degree of skill such as the movements performed in WL. In support, Kordi and Siahkohian [27] showed significant decreases in the competitive weightlifting exercises of the snatch (−6.0%) and clean and jerk (−5.1%), as well as the power snatch (−5.4%) and power clean (−6.6%) derivatives after just 2 weeks of detraining in elite WL athletes. Additionally, lower body strength also demonstrated significant reductions as indicated by the front squat (−5.9%) and the back squat (−5.8%), which in this case was similar to the reductions observed in the WL movements (i.e., −5.1% to −6.6%). Lean body mass also decreased (~0.9%) and body fat increased (~11.3%) over this period, although it is not clear if and how this contributed to the reductions in strength and power performance. A similar effect to that in Kordi and Siahkohian [27] has also been reported in a meta-analytical review by Bosquet et al. [28] during the first few weeks of training cessation. However, maximal force capability was more severely impacted with longer durations of training cessation (e.g., 16 weeks) compared to power [28]. To our knowledge, specific evidence about the detraining responses in PL athletes is not available. However, the results by Kordi and Siahkohian [27] regarding the reduction in front- and back-squat performance suggests that PL specific strength movements will also be affected with periods of ceased or reduced training. In further support, maximal strength and muscle cross sectional area have also been shown to be substantially reduced following 8–12 weeks of training cessation in well-trained adult males [29,30], which may be more akin to the training status of PL athletes compared to moderate or recreationally trained individuals. That being said, well-trained and younger athletes may be at an advantage when compared to novice individual and master’s athletes. For example, Bosquet et al. [28] also reported that the magnitude of detraining effect was larger for inactive individuals compared to athletes and those over 65 years of age. 

Although performance reductions during this period are likely, the available research suggests that muscle strength and quality may remain above basal levels (i.e., non-trained state) for substantial periods of time (Figure 1, Table 1). For example, Sakugawa et al. [31] suggest that elderly men and women maintain strength above baseline levels following 16 weeks of detraining. Although this may not directly reflect PL and WL athletes per se, we do acknowledge growing participation in each sport by Masters’ and/or elderly athletes. Further evidence suggests that this period may be substantially longer across different demographics. For example, lean mass and upper- and lower-body strength remain elevated after 24 weeks of training cessation in young men [32]. In addition, Ivey et al. [33] have reported that muscle quality (i.e., the amount of force produced per unit of muscle mass) can remain above baseline for 31 weeks following training cessation in young men and women and older men, respectively. Maximal dynamic strength has also been shown to remain above baseline for a similar period of time in young adult (21.4 ± 1.4 year) women [34]. However, Melnyk et al. [35] reported that quadriceps muscle cross-sectional area returned to baseline after a similar period in young and older males and females. Thus, although the available research is not specifically derived from strength athletes, it provides at least some reassurance that muscle strength, quality and mass will not diminish entirely following significant periods without training (Table 2). Further, functional losses may be attenuated with minimal training frequencies, for example see [36], and is discussed further in Section 3.1.

### 3.1. Minimum Training Frequency to Attenuate Performance Loss

It is now becoming more common that athletes have home access to equipment (e.g., lifting platform, squat racks, barbells and weight plates). If access to equipment is available, strength levels can potentially be maintained with minimal training frequency. This is important because although many individuals are currently isolated at home, juggling work and family duties may not always be conducive to more training opportunity. Importantly, although neuromuscular performance may temporarily improve following an acute period of reduced training (i.e., “taper”) (for example see Izquierdo et al. [26]), the current global pandemic has already lasted several months and may continue to impose restrictions on training facilities (e.g., number of patrons allowed or length of training sessions) for some time yet.

**Figure 2:**
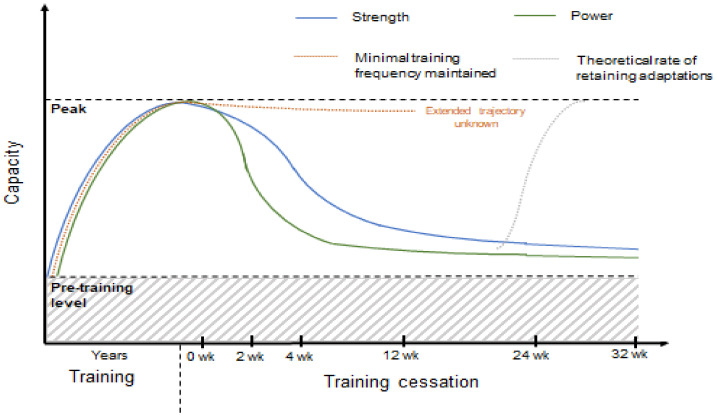
Theoretical depiction of the time-course of training cessation and possible retraining effects, based on available evidence from resistance training literature. “Baseline” refers to pre-training level of athletes’ neuromuscular capacity, “peak” refers to capacity prior to training cessation period. Orange dotted line indicates general neuromuscular (i.e., strength and power) capabilities of athlete.

A handful of studies have demonstrated that performing resistance training once or twice per week can minimize the loss of cardiorespiratory function [36] or, more specifically, maintain [37,38] or improve maximum strength [39] in some populations. Specifically, after an 8-week strength training period Tavares et al. [38] reported that half-squat one-repetition maximum strength and quadriceps cross sectional area were maintained when performing either 1 or 2 training sessions per week over a subsequent 8-week detraining period (exercise regime: 3–4 sets of 6–12 RM half-squat and knee extension exercise) when compared to ceasing training entirely. Similar effects have also been shown for other muscle groups [40]. For example, Tucci et al. [40] reported that following 10–12 weeks of initial training, isometric lumbar extension strength was maintained when resistance training was performed once per fortnight, or as little as once per month, but diminished if training was aborted entirely. (Refer to Figure 2, Table 2).

Collectively, the evidence presented above provides training-related information that can be used by and modified for strength athletes during the current and ongoing period of restrictions and training facility closures. Due to the complexity of programming and individualization of programs for competitive athletes, this information intentionally does not include suggestions about intrinsic session variables (e.g., volume, intensity and exercise selection), and thus, PL and WL coaches should aim to adapt current programs based on access to equipment. It appears that although a substantial loss of strength and power may be expected following several weeks to months of training cessation, some degree of confidence should arise that strength and muscle integrity can be maintained above baseline levels with significant periods of no training (e.g., up to 31–32 weeks). However, if access to appropriate training equipment is available, performing resistance training 1–2 times per week or potentially as infrequently as once or twice per month may attenuate a significant loss of neuromuscular capacity.

### 3.2. Can Strength Athletes Expect a Greater Rate of Re-Adaptation?

Despite ongoing debate and conjecture [41], recent opinion suggests that muscle may “re-adapt” at a quicker rate in previously trained individuals, colloquially termed “muscle memory”. Although the currently available evidence for this effect stems largely from animal models [42], this concept has, at least anecdotally, been widely discussed and reported by coaches and athletes. Specifically, the ability for greater muscle re-adaptation has been linked to mechanisms within muscles cells (e.g., increased nuclei number, altered gene expression and cell signalling) which persist even during substantial periods of detraining or complete cessation) [42,43,44,45,46,47,48]. For example, Egner et al. [45] demonstrate that despite muscle volume decline in mice, nuclei formed during training were still evident 3 months later; however, other examples suggest that this effect may last for years [46]. It is postulated that the preservation of myonuclei could then be used to facilitate retraining adaptations [47]. Evidence for retraining effects are presented by Lee et al. [48], who showed that myonuclei in mouse muscle are retained during 20 weeks of detraining, while muscle cross sectional area increased by a greater amount (6.9%) during 8 weeks of retraining compared to mice who performed the same exercise but were previously untrained.

In humans, limited work has directly investigated retraining effects, and the work that has been done has often examined the response with elderly men and women [49,50]. More recently, the retraining effect has also been investigated in young untrained adults [51]. In a unilateral leg strength training model, strength increased after a 5-week retraining period similarly for the trained and untrained leg. However, the authors acknowledge that the muscle memory hypothesis could not be ruled out as increases in myonuclei number were not observed during the initial training period. As highlighted by the authors, the mechanisms of muscle memory are not fully elucidated (e.g., potential neural contribution); thus, we suggest that using the opposite leg as a control may be a confounding factor due to potential cross-over effects. Further, support for a muscle memory effect in humans comes from Ogasawara et al. [52] who showed that rates of muscle and strength re-adaptation occurred faster when subsequent 6-week training blocks were separated by 3-week non-training phases in the bench press exercise. This finding has also been supported by the authors earlier work [22], and importantly, the amount and overall magnitude of the adaptation was not dissimilar to 15 weeks of continuous resistance training. Although limited evidence exists looking at this phenomenon with human subjects, the collective animal and preliminary human evidence provides support that previously trained strength athletes may undergo a greater rate of muscle adaptation once training is resumed (for theoretical depiction refer to Figure 1). However, to our knowledge, this has not been investigated in well-trained strength athletes and so further research is required to reach a definitive consensus and to provide well-supported evidence-based recommendations.

## 4. Overcoming Injury

Athletes who compete in strength sports are not immune to issues related to over-training and injury. In PL and WL, injury rates are similar to those in other non-contact sports requiring the expression of high amounts of strength and power [53]. PL and WL athletes report 1.0–4.4 and 2.4–3.3 injuries per 1000 training hours, respectively [53]. Of these injuries, approximately 26–33% occur in the shoulder, 26–31% occur in the hip, and 23–42% occur in the lumbopelvic area for men and women, respectively [53]. In WL athletes specifically, these values are somewhat similar: shoulder (36%) and lumbar region (24%) [54]. Thus, given that repetitious movements are performed under high training loads and intensities and large ranges of motion [55] by PL and WL athletes, a balance between training load and recovery is required [56]. In many sports, designated periods of the year (i.e., off-season) allow for prolonged recovery and incorporate well-structured calendar/season phases. However, in strength sports, this structure may not be as apparent with a plethora of local, national and international competitions available to compete in across the calendar year. 

Although providing specific evidence and professional recommendations for the many types of injuries suffered by strength athletes is outside the scope of the paper, we suggest several general strategies be considered. Specifically, these include consulting relevant professionals (e.g., physiotherapists) via in person or telehealth consultation, identifying causes of persistent/chronic injuries and focusing on rehabilitation strategies (e.g., retraining movement patterns) to overcome these issues prior to the resumption of full training and competition. In support, previous research has identified that many injuries in PL are associated with poor lifting technique under load [49]. Moreover, factors such as range of motion, or lack thereof, have been shown to correlate with squat depth [56], which is important for proper and successful skill execution in both PL and WL athletes. Thus, exercises to increase ankle and hip range of motion and dorsiflexor strength have been recommended for athletes where squat depth is restricted [57]. Additionally, poor shoulder mobility may also result in compensatory muscle actions and often contribute to injury in WL athletes [54]. Thus, increasing shoulder stability and flexibility may help attenuate these injury risks in WL athletes [58]. The current situation presents an opportunity for athletes to work with physical therapists and coaches (in person or via video assessment) to determine whether movement patterns are sub-optimal or limited by poor range of motion and, if so, attempt to correct these using decreased loads and/or targeted flexibility training. Furthermore, if a suboptimal movement pattern is identified, the athlete and medical professional should also aim to determine if the problem is an adaptative or maladaptive response to pain and discuss likely causes and solutions [59] (refer to Table 3).

## 5. Goal Setting and Planning

Effort towards effective goal setting for individual athletes has been well documented elsewhere (see [60,61] for examples) and so in this section we present a brief overview of information and applicability to strength athletes. Generally speaking, previous research has identified that goal setting strategies are adopted by most athletes in an attempt to improve performance, and overall, this process is considered moderate-to-highly effective [62]. Of these goals, both process (e.g., training related) and outcome (e.g., performance/competition related) goals are commonly implemented. For strength-specific sports, the adoption and adaptation of specific process and outcome goals as relevant to the experience and level of the athlete should be considered by the individual and coach. It is suggested that these are implemented in an attempt to improve both training-related and competitive aspects of PL and WL.

Although major shutdowns currently remain in place in any countries, the easing of restrictions is likely to see the gradual return of some, if not many, competitions in various countries. Therefore, PL and WL athletes may wish to consider the inclusion of both specific short- (e.g., process related focusing on training modification (see previous section and psychological considerations)) and long-term goals. In support, longer-term planning is feasible once competition scheduling resumes as such information is often available up to 12 months prior, with major national and international events usually occurring on similar dates each year. A further reason for this suggestion is that competitive participation in PL and WL can span over a large portion of the lifespan (e.g., junior, open and master’s categories). Although no known studies have tracked PL or WL athletes over their entire lifespan, recent evidence indicates that the mean length of time between an athlete’s first and final competition (over a 15-year analysis period) was 642 days and 582 days for male and female PL athletes, respectively [63]. However, this may be up to 3171 days (~9 years) for males and 2983 days (~8 years) for females [63]. Furthermore, how frequently an athlete competes has also been shown to influence maximum strength performance (refer to Pearson et al. [20]). Therefore, based on the current literature and organization of competitive strength sports, we suggest that PL and WL athletes strategically identify and plan for future competitions that (1) consider athlete experience, (2) assist competitive strategy (e.g., those required to qualify for subsequent national and international competitions) and (3) allow for training and performance improvements via appropriate temporality.

## 6. Psychological Considerations for the Competitive Strength Athlete

Apart from physical and physiological development, psychological factors can also contribute to overall athletic performance. In particular, individual strength-sport athletes may suffer from the negative impacts of anxiety during competition [64], which may be exacerbated compared to team-sport athletes [65]. In turn, this can potentially lead to a reduction in performance, and thus, PL and WL athletes should direct efforts toward identifying and improving psychological aspects of performance. In other largely individual sports, Mamassis and Doganis [66] showed that the practice of goal setting, positive thinking and self-talk, concentration and routine, arousal regulation techniques and imagery improved self-confidence and overall performance of junior tennis athletes. In fact, at the elite level, research has identified that gold medal winning Olympic athletes implement a number of mental training techniques including focus cues, self-talk, goal setting, imagery and relaxation [67]. Furthermore, significant differences have been reported between medallists and non-medallists [68], with emotional control was greater in medallists, while imagery was greater in non-medallists. The collective evidence provides support and rationale for the use of such techniques as a training tool in strength athletes. Further research has also identified that a high level of sport confidence positively influences an athlete’s thoughts and behaviours [69]. Thus, during periods of reduced or ceased physical training, strength athletes should place emphasis on the improvement of non-physical aspects of performance. Overall, PL and WL athletes should seek to become familiar with and implement mental strategies into modified training routines with the view towards facilitating future competitive performance.

## 7. Where to Next: The Importance of the Return of Strength Sports

### 7.1. Awareness of Physical and Mental Benefits 

**Figure 3:**
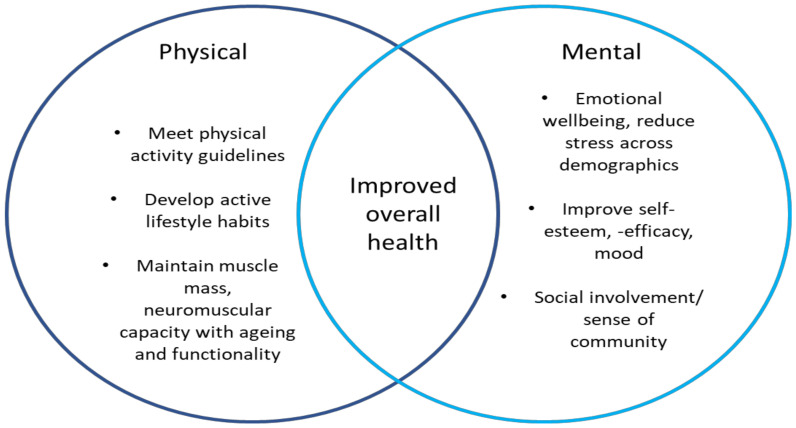
General and proposed benefits for athletes’ overall health (e.g., physical and mental) with strength sport participation.

In many sports, the benefits of participation extend far beyond the competition arena, positively influencing a number of health-related outcomes and behaviours [70]. In a physical sense, organized sport participation results in a greater likelihood of young adults meeting the physical activity guidelines [71], and thus, these individuals may develop a lifetime of involvement and, consequently, achieve an adequate amount of physical activity. In particular, both WL and PL are conducive to participation for young adults with junior classes now widely available to compete in. At the other end of the spectrum, master’s categories are also available and growing in popularity. Such categories offer a unique opportunity for sports participation with an equal playing field (e.g., athletes compete based on closely controlled age categories) that may not always be so well controlled in other sports. Moreover, the importance of muscle mass and strength to maintain health and function in latter adult years is also well-documented [72,73,74]. Thus, PL and WL (where training is dedicated to improved neuromuscular capacity) also provide an ongoing opportunity to support healthy ageing, independence and reduce mortality rates (see Figure 3).

Besides known physical benefits, it is also reported that sport participation can improve mental health and development [75], with this effect occurring across multiple domains and demographics. For example, Kelinske et al. [75] has shown that both males and females perceived sport to be beneficial for moral reasoning and socialization. In young adult females specifically, sport participation is also considered a moderate and positive predictor of self-esteem [76]. Moreover, participation in sport also improves psychological measures of self-efficacy, self-concept and self-esteem; mood and the locus of control in athletes with disabilities [77]. Importantly, PL supports participation in specialized competition for athletes with various disabilities (e.g., impaired vision, paraplegia) from the local to the international level. In addition, although team-based sports are often associated with improved psychosocial and physical activity related factors due to the social aspect of involvement, individual sports (e.g., PL and WL) can still improve mental health through self-awareness and personal growth [78]. Alternatively, Shores et al. [79] suggest that there is no difference in health behaviour across different sports, and although PL and WL specifically were not reported, some individual sports (e.g., surfing, snowboarding and skateboarding) were analysed in the study. Furthermore, Steptoe and Butler [80] demonstrate that active sport participation and vigorous activity positively associate with emotional wellbeing regardless of sex, social class and health status. Additionally, evidence also suggests that increased sports participation shows an inverse relationship with stress and distress [81]. In particular, these observations have been observed in unemployed mid-aged adults and unemployed young adults [81]. This is an important concept in the current global climate given the increase in individuals temporarily stood down from work or who have become unemployed since the onset of the pandemic. The latter is also an important consideration as the required costs to partake in PL and WL training and participation (e.g., personal equipment and registration fees) may be somewhat lower than other sports.

Despite numerous positive benefits, it is important to acknowledge that there can also be negative physical impacts observed at the elite sporting level. In particular, this is demonstrated by a prevalence of eating disorders, depression, distress and anxiety that can exist in such athletes [82]. Specifically, several authors have highlighted that eating disorders are more prevalent in sports where specific bodyweights or leanness is required [83,84,85]. Therefore, this risk should be acknowledged in elite WL and PL athletes who regularly implement strategies to compete in certain weight classes. Despite this, the large body of evidence, some of which has been presented above, suggests that competitive strength sport participation is likely to result in numerous physical and psychological benefits across competition levels. In particular, novice PL athletes made up ~63% of the sample of competition entries analysed in the study by Latella et al. [13] and so a vast majority may not be prone to such negative effects. Thus, the benefits of both recreational and elite strength sport participation should be promoted and emphasized by training facilities/clubs and sporting organizations as physical activity and social restrictions begin to ease (Figure 3).

### 7.2. Strength Sports as Communities

The value of sport and sporting participation to the community is recognized by the vast majority of local and national governments with many of the benefits noted in earlier sections. Sport, at least recreationally, may also foster a sense of community that is introduced by the programs and services offered and occurring within facilities [86]. Although WL and PL are technically individual competitor sports, athletes often train at specialized gymnasiums or form part of a representative team (i.e., club, state or national) at major competitions. Thus, training and competition for strength sports also offers a novel and unique opportunity to engage with other like-minded members of the community and form a community in itself. However, this opportunity has been severely impacted in a number of counties for several months and is continuing to varying degrees. Therefore, we suggest that training clubs and organizations seek to develop unique strategies that foster a sense of community for the collective individuals (i.e., athletes and coaches) involved. Such approaches may be derived from recent telehealth initiatives [87] and adapted to suit strength athlete training programs, promote online socialization and “check-ins” with other fellow athletes and maintain a sense of community within or between training facilities.

Collectively, evidence suggests numerous physical and mental health benefits from sport participation, and this is likely to extend to PL and WL settings. These benefits may occur irrespective of age or sex and, in particular, some such as socialization, sense of community and stress reduction may be of particular relevance given the current and ongoing global situation. Thus, we encourage WL and PL athletes to consider the extended benefits of regular training and competition in each respective sport. Moreover, we also encourage PL and WL clubs and organizations to emphasize and promote these benefits to athletes alongside more traditional athletic goals and work toward new initiatives to foster a sense of community and ongoing participation or a return to it. 

## 8. Conclusions

The current global pandemic presents many challenges, including those faced by sporting organizations, professionals and athletes. For strength athletes, specific recommendations are required due to the dissimilar performance requirements compared to team- and field-based sports. Based on the available evidence, it appears that a significant loss of muscle strength, power and mass can occur, beginning within weeks of the final training session. However, these variables are likely to remain above basal levels for many months, and loss may be attenuated (completely or partially) with training frequencies of 1–2 times per week or less. Additionally, we also suggest that this time be used to overcome persistent injury, develop short- and long-term goals and implement psychological training strategies to assist future competitive performance. Moving forward, the reopening of training facilities and the reinstatement of strength sport competitions also have extended benefits for individual and community health. These include athlete’s positive physical, mental and psychosocial wellbeing, some of which may have particular relevance to the current and ongoing global situation. We therefore suggest that these benefits also be promoted by gymnasiums and strength sport organizations to encourage participation. It is intended that these recommendations may be adopted and adapted by coaching professionals and strength athletes where competition or access to training facilities and equipment is limited or non-existent due to ongoing and future restrictions.

## Figures and Tables

**Table 1:** Number of documented competitor entries, regardless of organization, for each country from the 1 January 2020 to 2 May 2020 compared to same dates in 2019 (sourced from [21]). Countries where results were not available for both years or were deemed incomplete after manual inspection were omitted.

	Competitors: Year to Date (n)	
Country	2019	2020
USA	26,945	20,620
Russia	4276	118
United Kingdom	3625	1750
Australia	1951	951
Japan	1200	167
Italy	921	557
Norway	766	371
Slovakia	698	60
New Zealand	466	78
Canada	424	122
Sweden	417	312
Austria	360	38
Spain	306	272
Netherlands	235	203
Portugal	180	126
Brazil	160	33
Switzerland	147	54
Belarus	100	30
Belgium	71	35
Finland	60	7
Thailand	52	31

**Table 2:** Brief overview and summary of main findings regarding neuromuscular outcomes from reported studies. Studies examining both reduced training frequency and training cessation concurrently are documented in one section only. CSA: cross sectional area, 1-RM: one-repetition maximum, IEMG: isometric electromyography, LM: lean mass, RTD: rate of torque development, wk: week, y: year

Study	Participant Characteristics	Study Protocol	Summary of Main Findings
**Training Cessation**			
Ogasawara et al. [22]	Untrained men, 24.7 ± 2.5 y (n = 15)	Bench press training (3 days p/wk) 15 wk continuous OR 6 wk then 3 wks no-training followed by 6 wks retraining.	No significant decreases in muscle CSA and 1RM after 3 wks of training cessation.
Tran et al. [23]	Competitive surfers, 14.1 ± 1.6 y (n = 19)	4 wks strength training cessation but maintained surfing participation.	Decreased vertical jump height (−5.3%), vertical jump peak velocity (−3.7%), isometric strength (−5.5%), relative isometric strength (−7.3%) and sensorimotor ability (i.e., athletes took longer to stabilize from a dynamic landing task).
McMaster et al. [24]	Elite rugby union, rugby league and American football athletes	Systematic review article.	Strength levels maintained for up to 3 wks after cessation, but rate of decline increases between 5–16 wks.
Chtourou et al. [25]	Healthy male physical education students, 23.1 ± 1.9 y (n = 31)	14 wks strength training (squat, leg press, leg extension, leg curl) 8–10 RM then 5 wks of no training.	Squat jump and maximal voluntary contraction partially retained after 3 wks, but lost after 5 wks.
Izquierdo et al. [26]	Basque ball playing men, ~24 y(n = 46)	16 wk periodized training followed by 4 wk training cessation or taper.	Decrease in maximal strength (−6 to −9%) and muscle power output (−17 to −14%) of the arm and leg extensor muscles. Greater decrease for power compared to strength.
Kordi and Siahkohian [27]	Elite male weightlifters (n = 12)	2 wks training cessation.	Decreased snatch (~12kg), lift and jerk (~12kg), back squat (~10kg), front squat (~9kg), power snatch (~7kg).
Bosquet et al. [28]	Mixed training status, sex and age	Systematic review with meta-analysis.	Similar decrease in strength and power during initial weeks but greater decrease in strength with longer durations. Reductions greater in older people and inactive people for strength and power.
Hakkinen et al. [29]	Strength trained males, 20–32 y (n = 11)	24 wks strength training between 70 and 120% of maximum followed by 12 wks of training cessation.	Decrease in maximal strength which correlated with the decrease in maximum IEMGs of the leg extensors. Decreased mean muscle-fibre area (both fibre types).
Hakkinen and Komi [30]	Strength trained males, 26.4 ± 0.6 y (n = 14)	Concentric and eccentric strength training of leg extensors (80–120% of concentric maximum), 3 × p/wk for 16 wks followed by 8 wks of training cessation.	10.5% decrease in IEMG during first 4 wks of training cessation. Decrease in force of ~8.3% after 24 wks.
Sakugawa et al. [31]	Elderly men and women, 64.0 ± 2.3 y (n = 10)	12 wks of strength training, 16 wks of training cessation and 8 wks of retraining.	Maximum strength remained above baseline after 16 wks. Retraining recovered maximum strength gains, RTD and functional capacity.
Lo et al. [32]	Health men, 20.4 ± 1.4 y (n = 10 per group)	24 wks of strength or endurance training, followed by 24 wks training cessation.	Strength and LM greater than the baseline values after 24 wks of training cessation.
Ivey et al. [33]	Young men, 25 ± 3 y (n = 11); young women, 26 ± 2 y (n = 9); older men, 69 ± 3 y (n = 11) and older women, 68 ± 3 y (n = 11).	9 wks of strength training followed by 31 wks of training cessation.	Muscle quality remained elevated above baseline in all groups except for older women.
Staron et al. [34]	Females, 21.4 ± 1.4 y(n = 6)	20 wks lower-limb strength training followed by 30–32 wks of training cessation then 6 wks retraining.	Small effect on fibre cross-sectional area but increased percentage of type IIb fibres and concomitant decrease in IIa fibres. Maximal dynamic strength decreased but remained above baseline.
Melynk et al. [35]	Young males, 25 ± 3 y (n = 11); older males, 69 ± 3 y (n = 11); young females, 26 ± 2 y (n = 10); and older females, 68 ± 3 y (n = 11)	9 wks unilateral knee extension strength training followed by 31 wks of training cessation.	Muscle CSA was not different to baseline in older males and young and older females but remained above baseline in young males.
**Training frequency**			
Ronnestad et al. [37]	Professional male soccer players, 22–26 ± 2 y, (n = 14)	10 wk strength training (2 × p/wk) followed by one group performed 1 session p/wk, another group performed 1 session p/fortnight.	1 × p/wk training maintaining strength, sprint and jump performance. 1 × p/fortnight strength training reduced leg strength and 40 m sprint performance.
Tavares et al. [38]	Untrained males, 24.7 ± 3.9 y (n = 33)	8 wks of strength training (3–4 sets of 6–12 RM, three sessions/week in half-squat and knee extension exercises) followed by 8 wks reduced training, i.e., strength training 1× p/wk, 2 × p/wk or complete cessation.	No significant decrease in 1 RM and CSA with reduced training frequencies. However, a decrease in half-squat 1 RM (22.6%) and CSA (5.4%) was observed with complete training cessation.
Androulakis-Korakakis et al. [39]	Healthy men, ≥ 1 year of strength training experience	Systematic review with meta-analysis.	Minimum of 1 set 1 × p/wk may improve strength. Unclear if similar effect in highly trained strength athletes.
Tucci et al. [40]	Trained males, 34 ± 11 y (n = 34); and females 33 ± 11 y (n = 16)	10–12 wks of lumbar extension strength exercise 1, 2 or 3 × p/wk followed by reduced training, i.e., 1 × p/fortnight (n = 18) or 1 × p/month (n = 22) for 12 wks.	Training 1 × p/fortnight and 1 × p/month showed no significant reduction in lumbar extension strength. Training cessation resulted in significant ~55% strength loss.

**Table 3:** Summary of evidence, general suggestions and recommendations for strength athletes during prolonged periods of reduced or ceased training.

Focus Area	Summary, Suggestions and Recommendations
Physical performance capacity	Avoid significant periods of no training (e.g., ≥ 2–4 weeks for power, 2–5 weeks for strength). Training 1–2 times per week may attenuate performance loss. Some strength may be retained with more infrequent training frequencies (i.e., once per fortnight or month). Some neuromuscular and muscular properties may remain above baseline for prolonged periods without training (e.g., up to 32 weeks).
Injury	Often full recovery is not allowed due to ongoing training and competition. Lower athlete load (less training and competition) may allow additional recovery. Consultation with relevant professional therapists (in person or telehealth) is suggested if required. Movement patterns and ongoing causes of pain should be identified for effective rehabilitation. Range of motion and stability should be improved if problematic for the individual athlete.
Goal setting and planning	This period can also be used to set or re-set goals. Both short- and long-term goals should be incorporated. Consider both process and outcome goals. Coaches and athlete should work together to identify appropriate goals that consider experience level of athlete.
Psychological considerations	Consider mental strategies to overcome competitive anxiety and improve self- and sport-confidence. Strategies may include goal setting, positive thinking/self-talk, concentration/routine, arousal regulation techniques, imagery, focus cues, self-talk, imagery and relaxation/emotion control. Can be used in the lead up to and during competition.
